# Evaluation of InTray Cassettes Directly from Blood Cultures for the Diagnosis of Sepsis in Clinical Bacteriology Laboratories as an Alternative to Classic Culture Media

**DOI:** 10.3390/diagnostics13030523

**Published:** 2023-01-31

**Authors:** Alessandra Natale, Saoussen Oueslati, Alice Rochard, Sien Ombelet, Daniel Lopez-Baez, Liselotte Hardy, Jane Cunningham, Céline Franquesa, Olivier Vandenberg, Jean-Baptiste Ronat, Thierry Naas

**Affiliations:** 1 Médecins Sans Frontières, Operational Center Paris, 75019 Paris, France; 2 Team ReSIST, INSERM U1184, Université Paris Saclay, CEA, Inserm, Immunologie des Maladies Virales, Auto-Immunes, Hématologiques et Bactériennes (IMVA-HB/IDMIT), 92265 Fontenay-aux-Roses & Kremlin Bicêtre, France; 3 Service de Bactériologie-Hygiène, Hôpital Bicêtre, AP-HP, 94270 Le Kremlin-Bicêtre, France; 4 Department of Clinical Sciences, Institute of Tropical Medicine, 2000 Antwerp, Belgium; 5 Hampshire Hospitals Foundation Trust, Basingstoke RG24 9NA, UK; 6 Access Campaign, Médecins Sans Frontières, 1211 Geneva, Switzerland; 7 Center for Environmental Health and Occupational Health, School of Public Health, Université Libre de Bruxelles (ULB), 1070 Brussels, Belgium; 8 Innovation and Business Development Unit, Laboratoire Hospitalier Universitaire de Bruxelles—Universitair Laboratorium Brussel, Université Libre de Bruxelles (LHUB-ULB), 1000 Brussels, Belgium; 9 Division of Infection and Immunity, Faculty of Medical Sciences, University College London, London WC1E 6BT, UK

**Keywords:** InTray cassettes, InTray^®^ Müller-Hinton (MH) chocolate, Colorex™ screen, bloodstream infection, diagnosis of sepsis, clinical bacteriology in low resource settings, polymicrobial infections, blood cultures, subcultures, LMICs

## Abstract

Culture media is fundamental in clinical bacteriology for the detection and isolation of bacterial pathogens. However, in-house media preparation could be challenging in low-resource settings. InTray^®^ cassettes (Biomed Diagnostics) could be a valid alternative as they are compact, ready-to-use media preparations. In this study, we evaluate the use of two InTray media as a subculture alternative for the diagnosis of bloodstream infections: the InTray^®^ Müller-Hinton (MH) chocolate and the InTray^®^ Colorex™ Screen. The InTray MH chocolate was evaluated in 2 steps: firstly, using simulated positive blood cultures (reference evaluation study), and secondly, using positive blood cultures from a routine clinical laboratory (clinical evaluation study). The Colorex Screen was tested using simulated poly-microbial blood cultures. The sensitivity and specificity of the InTray MH chocolate were respectively 99.2% and 90% in the reference evaluation study and 97.1% and 88.2% in the clinical evaluation study. The time to detection (TTD) was ≤20 h in most positive blood cultures (99.8% and 97% in the two studies, respectively). The InTray^®^ MH Chocolate agar showed good performance when used directly from clinical blood cultures for single bacterial infections. However, mixed flora is more challenging to interpret on this media than on Colorex™ Screen, even for an experienced microbiologist.

## 1. Introduction

Culture media are fundamental in any clinical bacteriology laboratory. Conventional culturing methods in clinical laboratories generally consist of using standard agar poured plates or commercially available pre-poured ready-to-use agar plates. However, ready-to-use plates are not adapted to the logistics of low-resource settings because of their short shelf life, usually under six months, or even less when containing blood components. Therefore, laboratories in low- and middle-income countries (LMICs) rely on dehydrated culture media and in-house preparation (rehydration of powder with sterile water). This is challenging, time-consuming and requires specific equipment, space (a dedicated autoclave for sterilisation and space for plating), well-trained personnel and a strong quality control system [1]. Moreover, the addition of sterile animal blood or specific substrates may be difficult to obtain or challenging to supply (short shelf-life, cold chain requirement, price). Centralised media preparation, acquisition, tropicalization and customised solutions by in-vitro diagnostics manufacturers are among the suggested solutions [1].

Bloodstream infection is the presence of an infectious agent in blood. If not treated properly, this can cause sepsis, a deregulated response of the body to the infection, subsequently causing life-threatening organ failure with negative outcomes. It has been estimated that the incidence of sepsis worldwide is 48.9 million, with 11.0 million sepsis-related deaths in 2017 [2]. LMICs are the most affected, in particular by sepsis-related deaths, with 8.2 million deaths estimated in 2017 (84.8% of the total) [2]. Sepsis can be treated, and deaths avoided with a timely and accurate diagnosis. To combat this important global health threat, the WHO responded with a WHO Secretariat Report and, in May 2017, the Seventieth World Health Assembly (WHA) adopted the WHA70.7 Resolution [3].

In the clinical diagnostic process, the detection of bloodstream infections starts with the sampling of a small volume of blood from the patient. After overnight incubation in the laboratory in a specific bottle with nutrient media (blood culture bottle), pathogens can hopefully be analysed by subculturing the positive blood culture on agar plates [4,5]. The type of culture media is important to facilitate the growth of pathogens. Usually, a combination of selective and non-selective media helps the technicians to predict the type of bacteria that were present in the original blood sample by grouping them by their general characteristics (Gram-positive or negative, bacilli or cocci etc.) [6]. This step is crucial to give clinicians an orientation for initial treatment [7] but also to help lab technicians choose the most appropriate tests required for full pathogen identification and antibiotic resistance patterns. The capacity to recover the pathogen, plus time to results and accuracy are therefore important quality indicators in the first step of the process. Beyond the clinical relevance at the patient level, blood culture data analysis can further shed light on microbiological surveillance and antibiotic stewardship, both essential components of the WHO action plan to contain antibiotic resistance [8].

Bloodstream infections are generally caused by a single bacterial pathogen, but co-infections with multiple pathogens may occur. The causative agents include gram-positive and gram-negative bacteria, anaerobes, fungi, and parasites, depending on settings, patient population, infection entry site etc. [9,10]. Polymicrobial infections might occur, depending on the patient population, in 1–2% of patients, particularly with severe immunodeficiency and with a polymicrobial focus such as abdominal (perforation, abscesses) and gynaecological infections [11]. MSF internal data from conventional microbiology laboratories in low-resource settings show that in these locations, mixed infections can reach 10% of cases, especially in pediatric populations.

Within the context of the Mini-Lab project, an MSF initiative to realise a small-scale, transportable, clinical microbiology laboratory adapted to low-resource settings [12,13], we tested whether an alternative subculturing solution, the InTray™ cassettes (BioMed Diagnostics, Inc., White City, OR, USA), could be used for bloodstream infection diagnosis directly from blood cultures. These in-vitro culture-media cassettes have several advantages: FDA and CE-marked, ready-to-use, small size for easy transport and storage and long shelf-life even with blood-containing media (12 months for most agar formulations). They can also be customised with nearly any media and are recommended for the isolation, cultivation and counting of fastidious and non-fastidious organisms [14,15]. For this evaluation, firstly, we performed a proof-of-concept study with the following objectives: (a) test the use of InTray cassettes directly from blood cultures, and (b) select the most convenient media for our application. Then, we evaluated the two selected InTray media compositions; the InTray MH (Muller Hinton) chocolate agar for the detection of the most frequent pathogens causing bloodstream infections and the InTray Colorex Screen for the detection of polymicrobial infections. The selected media were evaluated in two different studies in two European laboratories: a reference validation study at the Institute of Tropical Medicine in Antwerp and a clinical evaluation study at the microbiology and hygiene laboratories at Bicêtre University Hospital.

## 2. Materials and Methods

### 2.1. Proof-of-Concept Study

To test the use of InTrays cassettes directly from blood cultures and to select the media composition best suited for our application, we previously carried out a small proof-of-concept study. Briefly, 4 different formulations of InTray^TM^ cassette media were compared (Tryptic Soy Agar -TSA-, Tryptic Soy Agar with 5% sheep blood, Mueller Hinton -MH- Chocolate Agar, and Colorex Screen) to 3 respective reference media: Columbia agar with 5% sheep blood and the Chocolate agar with PolyViteX (bioMérieux, Marcy-l’Étoile, France), and UriSelect™4 (Bio-Rad Laboratories, Marnes la Coquette, France). A total of 30 blood cultures were directly inoculated on all types of InTray cassettes and reference media. Half of these (n = 14) were clinically positive blood cultures retrieved from a clinical laboratory, and 16 were simulated positive blood cultures (prepared as further described in the clinical evaluation study).

### 2.2. Reference Validation Study

A total of 490 BacTAlert bottles (bioMérieux), BacT/ALERT FA Plus Aerobic and BacT/ALERT PF Plus were used to evaluate the performance of InTray MH chocolate agars, compared with reference chocolate agars (Becton-Dickinson, Franklin Lakes, NJ, USA) at the Laboratory of the Institute of Tropical Medicine (Antwerp, Belgium). The protocol for the preparation of the simulated blood cultures and processing of positive cultures has been described elsewhere [16,17,18]. A selection of 20 species was inoculated in the blood cultures. These strains were isolated during research studies based in different tropical or LMICs like the Democratic Republic of Congo (DRC), Ecuador, Benin, Burkina Faso, Cambodia, Gambia, but also France and Peru (Appendix C, Table A2). For the subculturing of the InTray cassettes, an optimised subculturing protocol (Appendix A) was used. After inoculation, InTray cassettes were placed in a normal ambient incubator, and reference media were incubated in a normal ambient incubator for all non-fastidious organisms and in a CO_2_ incubator for fastidious organisms, both at 35 ± 1 °C. The choice of air atmosphere for the incubation of InTray cassettes independently from the type of organisms was justified by a small proof of concept study (Appendix B). Reading was done after 16 h or up to 48 h if no detection of growth was detected earlier. Data collection and analysis were the same as described elsewhere [16].

### 2.3. Clinical Evaluation Study

During the study period between November 2018 and March 2019, 70 positive BacT/ALERT FA Plus blood cultures, as confirmed by a positive signal by the automated detection system and by the clinical laboratory, with less than 7 days from date to positivity were retrieved from the clinical laboratory of the Bicêtre University Hospital (Le Kremlin-Bicêtre, France). The 70 blood cultures corresponded to 70 patients, of which 65 were adults and 5 were paediatric patients. The spectrum of organisms retrieved in this context was mainly composed of *Staphylococcus aureus* (30%) and coagulase-negative Staphylococci (CNS; 25.7%; Table 1). For the subculturing of the InTray cassettes, the optimised subculturing protocol of the reference validation study was used (Appendix A). After inoculation, according to routine clinical laboratory results, InTray MH chocolate cassettes were placed in a conventional air incubator, and reference media were incubated in a conventional air incubator for all non-fastidious organisms and in a CO_2_ incubator for fastidious organisms at 35 ± 1 °C. Reading was done after 16 h or up to 48 h if no growth was detected earlier. Data and pictures were collected and analysed with Excel (Office365, Microsoft Corporation, Washington, DC, USA).

### 2.4. Detection of Poly-Microbial Cultures

For the detection of polymicrobial cultures, simulated blood cultures were inoculated with a mix of 2 bacterial species prepared in a saline solution. The procedure was as follows. Each clinical isolate was first plated onto standard chocolate agar for colony counting. Then dilutions from fresh subcultures of both strains were mixed in a saline solution. A volume of 100 µL of the mix was inoculated together with 2 mL of human blood into a blood culture bottle to obtain a final bacterial concentration of 5–50 CFU/species/bottle. The same volume of saline solution mix was also directly plated onto reference media (UriSelect™4) to check growth prior to incubation. After overnight incubation in a normal air incubator at 35 ± 1 °C, one drop of the blood culture was inoculated onto InTray MH chocolate agar, InTray Colorex Screen and reference UriSelect. The optimised subculturing protocol (Appendix A) was used. In total, 11 types of mixes were prepared, divided into 3 main categories: clinically relevant mixes (as by MSF internal data), colour challenge mixes, including species colonies with an expected similar colour, and contaminant mixes, including a pathogen and a contaminant (Table 2). Each mixture was prepared using 3 different stains of the same species and plated in triplicate on each media (for a total of 99 inoculated mixes).

In all studies, sensitivity was calculated as the number of plates with any growth over total inoculated plates, while the specificity at the species level was the number of species with “expected growth characteristics” on InTray over the total species analysed. In “discrepancy of growth,” we included a slower growth or other growth variations, like colony colour, colony aspect etc., compared to the same species on reference media (“expected growth”). The ease of use of the InTray cassette was evaluated using the possibility of picking single colonies and the identification of polymicrobial cultures on the agar. Feedback on the usability of the cassettes was also collected from the users.

## 3. Results

### 3.1. Proof-of-Concept Study Results

After 16 h, the incubation recovery rates on InTray cassettes were: 24/30 on TSA, 27/30 on TSA + 5% sheep blood agar, 28/30 on MH chocolate agar, and 23/30 on Colorex Screen, compared to 27/30 on the reference Columbia + 5% sheep blood, 29/30 on chocolate agar with PVX, and 23/30 on UriSelect media. The difference between the InTray MH chocolate agar and the reference chocolate agar with PVX was due to the lack of growth of one strain of *Streptococcus* spp, a fastidious organism. The 2 main conclusions of this proof-of-concept were that; (a) overall, the InTray cassettes were comparable to reference media for subculturing directly from blood cultures; (b) the best InTray media for recovering a wide range of pathogens from mono-microbial cultures and for detection of poly-microbial cultures were the InTray MH chocolate and the Colorex Screen, respectively.

### 3.2. Reference Validation Study Results

In this study, out of a total of 490 blood culture bottles inoculated with 20 bacterial species, 486 grew on the InTray MH chocolate agar after 24 h incubation, while all 490 grew on the reference media. The only two species that showed growth discrepancy between the InTray MH chocolate and the reference media were *Streptococcus pneumoniae* and *Streptococcus pyogenes*. In this study, the sensitivity was, therefore, 99.2% (CI 95%; 98.3–99.9%). Also, only two out of 20 species showed a growth variation (suboptimal growth) on InTray cassettes compared to the reference method, giving a specificity of 90% (CI 95%; 76.8–100%).

Additional testing of several *S. pneumoniae* and *S. pyogenes* strains showed that a longer incubation time of the InTray cassettes (up to 48 h) and incubation in a conventional incubator instead of in a CO_2_ incubator improved the growth of the two *Streptococcus* species. Extended incubation time (>24 h) improved growth for *S. pneumoniae* but not for all *S. pyogenes* tested: out of a total of 12 *S. pyogenes* strains tested, 11 showed good growth after 48 h, indicating that growth variation was strain-specific more than species-specific (Table 3).

The time to detection in this study was, therefore, 24 h for 99.2% of cultures and 48 h for all the strains except for 1 *S. pyogenes* strain. Furthermore, the presence of single colonies occurred in 86% of the InTray cassettes compared to 99% of the reference media, with more than 10 usable colonies for downstream analyses in 31% of cases compared to 68% for the reference method. The median was five colonies (min = 0, max = 48) for the InTray and 15 (min = 0, max = 214) for the reference media.

### 3.3. Clinical Evaluation Study Results

In this study, 70 positive blood cultures were inoculated on the InTray MH chocolate agar and on the reference agar plates. No difference in terms of positivity was observed between the InTray cassettes and the reference media, with a concordance of 100%. The sensitivity of the InTray cassette was 97.1% with any growth, as it was for the reference. In both cases, 1 strain of *Streptococcus pneumoniae* and 1 of *Streptococcus constellatus* did not grow on the culture media (Table 4).

Time to detection was 16 h in 81% of cultures and 20 h in 100% of cultures on both media. The slowest strains to grow were one *Micrococcus luteus*, one *Staphylococcus aureus*, one *Streptococcus mitis*, one *Staphylococcus pettenkoferi*, two *Staphylococcus capitis*, and five *Staphylococcus epidermidis* strains. Strains with variation compared to the reference are reported in Table 5.

Sensitivity and specificity at the species level on the InTray MH chocolate were 97.1% (CI 95%; 93.2–100%) and 88.2% (CI 95%; 72.9–100%), respectively, as two species out of the 17 inoculated showed a discrepancy with growth observed on the reference media. In this study, of all the species successfully cultured on InTray cassettes, a difference in growth characteristics was observed in two species, *Micrococcus luteus* and *Staphylococcus epidermidis*; in the first case, the colonies were of considerably smaller size than on the reference media, while in one strain of *S. epidermidis* the overall growth was very weak on InTray cassette opposite to the reference media, even after prolonged incubation (Table 5). Isolated colonies were available on 62 InTray cassettes (62/68, 91.2% of grown cultures), with more than 10 isolated colonies available on 36 of them (36/68, 53% of grown cultures), compared to 100% on reference media.

### 3.4. Detection of Polymicrobial Cultures Results

Of the 33 total mixed cultures (11 mixes, each in triplicate) inoculated on both InTray MH chocolate agar and on InTray Colorex Screen, 100% showed any growth both on Colorex Screen and on MH chocolate agar. However, a clearly detectable mixed culture was detectable in 17 of the 33 mixes inoculated on Colorex Screen (51.5%) and only in 8 of the 33 mixes (24.2%) of MH chocolate agar.

All the strains classified as “contaminants” (*Candida albicans*, *S. epidermidis* and *Corynebacterium striatum*) plus *S. pneumoniae*, in the mix with *E. coli* and with *K. pneumoniae*, had difficulty growing, both on InTray cassettes and on the reference media. Growth controls of the mix inoculated onto reference media before incubation in the blood culture bottle were always positive, and the two strains were clearly detectable. Including in the analysis only those mixes with both inoculated species were recovered from the reference media, a mixed flora was discerned on a total of 51 (94.4%) of 54 grown plates (CI 95%; 88–100%) on Colorex Screen, and in 24 (44.4%) of the 54 (CI 95%; 31.2–57.7%) MH chocolate agar InTray cassettes. 

**Figure 1:**
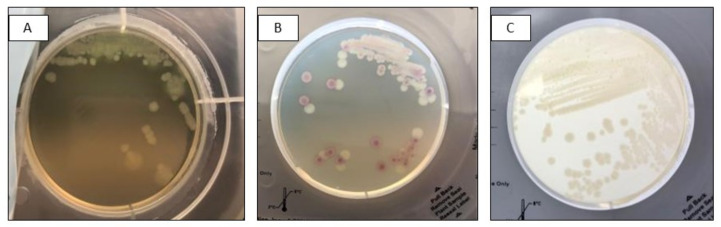
Mixed culture of *Salmonella* spp. (pink) and *Pseudomonas aeruginosa* (white) after subculture on InTray Colorex Screen (A) Visualization of mixed flora based on morphology; (B) Detection based on colony colour; (C) Mixed infection not detected.

However, these results were not consistent among replicates; for example, the combination of *Enterococcus faecalis* and *E. coli* was detected on Colorex Screen thanks to colour differentiation (*E. coli* pink and *E. faecalis* blue) but missed on 2/3 of replicates on InTray MH chocolate because *E. faecalis* colonies were too small. Again, only in one of the three replicates of mixes made with *Salmonella spp.* and *Pseudomonas aeruginosa*, the colonies of the two species were so similar in morphology and colour that it was not possible to distinguish among them, while in the other two mixes made of different strains of the same species, colour and morphology were sufficiently different to detect the mixed flora (Figure 1).

Of the total number of strains grown (including replicates, 9 × 18 strains = 162), the presence of single colonies occurred in 132 or 81.5% (95% CI; 75.5–87.4%) on InTray Colorex Screen compared to 100% on the reference media, while only in 67.9% (95% CI; 60.7–75.1%) on MH chocolate InTray cassettes (Table 6).

Of all the species grown on MH chocolate and on the Colorex Screen, the colour and aspect of colonies were comparable to their aspect on the reference media. Only in the case of *Acinetobacter baumannii* were colonies more difficult to recognize on the InTray MH chocolate than on the reference media (Table 7). Overall, the colonies were less defined and with more irregular margins on the InTray cassettes than on the reference media; nevertheless, they were recognizable by an expert microbiologist’s eye.

## 4. Discussion

The InTray cassettes have already been successfully used in LMICs, targeting uropathogens from urine specimens [19] and ESBL Enterobacterales from blood cultures, where the combination of Colorex Screen and ESBL chromogenic media was able to reduce turnaround time significantly [20]. In this study, we tested the combination of InTray™ MH chocolate and Colorex Screen media for a wider application, in early orientation and diagnosis of sepsis, as a simple alternative to the classic combination of selective and non-selective media. Given the variability of bloodstream infection pathogens, media characteristics were chosen to allow the detection of the widest possible range of pathogens using the smallest and easiest possible combination of media. Pre-identification tests, such as Gram staining and other simple biochemical tests (oxidase, catalase etc.), can be directly performed on single colonies on chocolate agar, while colony characteristics on Colorex Screen assist preliminary species identification and give additional information about mono and poly-microbial cultures. Early reporting has a high impact on the early selection of effective antimicrobials for patient treatments [7,21].

In both the reference validation and the clinical evaluation study, most of the organisms inoculated were able to grow on the InTray™ MH chocolate agar after overnight incubation in a normal atmosphere incubator. Overall, only *Streptococcus* spp. showed delayed or no growth on InTray™ cassettes compared to the reference media. However, these organisms are often called “fastidious organisms” because they require special conditions for optimal growth, such as a 5% carbon dioxide atmosphere [22]. Surprisingly, we found that the growth of these fastidious organisms on InTray cassettes was optimized by incubation in a normal atmospheric incubator instead of in a CO_2_ atmosphere. The InTray™ GC cassette with an integrated CO_2_ disk, appositely designed by the manufacturer for fastidious organisms [23], showed no advantage, in our experience, compared to regular cassettes incubated in a conventional incubator (Appendix B). This is actually advantageous, as the possibility to incubate “fastidious organisms” in a normal air incubator instead of under a CO_2_ atmosphere reduces equipment and makes laboratory workflow easier for non-expert microbiologists.

In fact, in the context of the Mini-Lab project in which this study was carried out, the ability of the laboratory to be operated by non-expert microbiologists was a key aspect. Therefore, during development, the diagnostic test selection was also based on ease of use. Because the diameter and depth of the InTray cassettes’ agar are reduced compared to standard agar plates (6 cm versus 9 cm in diameter, respectively), although media content remains similar [24], colony growth characteristics could be affected. Growth in a lawn may occur, obstructing the collection of single colonies for pre-identification tests and downstream analyses. Also, poorly discernible colony colour or morphology might delay accurate identification. In this study, single colonies were easily detectable on InTray™ MH chocolate agar when a single pathogen was responsible for the infection, but not in the case of a polymicrobial infection. In this case, instead, Colorex Screen showed well-defined and easily discernible single colonies, allowing recognition of polymicrobial growth. Colony characteristics and colours on Colorex Screen matched the reference media, facilitating its use for non-experts. On the contrary, on InTray™ MH chocolate, polymicrobial cultures were difficult to detect, and pathogens were not easily recognisable by colony morphology alone, even by an expert microbiologist. On Colorex Screen, only the types of mixed cultures using “contaminants” didn’t give the expected results. However, since missing growth also on reference media occurred after overnight incubation in blood culture bottles, we deduced that in the blood cultures, these species were over-competed and died off. We are aware that this was a limitation of this study. Interestingly, this occurred with all the species usually representing “contaminants” or skin flora; unfortunately, we didn’t perform any tests without incubation in blood cultures, as we wanted to recreate conditions as similar as possible to real samples. Other study limitations include a small sample size and lack of testing in a laboratory based in LMICs.

The use of the InTray™ cassette was favourably evaluated by the user with positive feedback, in particular on the safety provided by the resealable label with its anti-fog window, which also helps avoid spillover. However, two factors were identified as potentially impeding good isolation of colonies: the presence of condensation inside the cassette and the need for better control of drop size from blood cultures when dispensing the drop in order to avoid overgrowth of bacterial cultures. Protocols were optimised following up on this feedback (Appendix A and Appendix B).

However, before considering to implement diagnostics in LMICs, a consideration about costs is deemed necessary. In this case, a general consideration of costs could reveal that whilst initial medium costs are higher for InTray™ cassettes, when we account for other fixed and variable costs the final per unit cost of specimen and isolate might not be that different. In the context of the Mini-Lab project, a comparison should be made between costs traditionally incurring in LMICs settings using reference culture and Mini-Labs using InTray™ cassettes. This might show that the initial higher medium costs could be offset by lower initial set-up, maintenance, rent and labour costs when using conventional in-house media preparation. The degree to which this is offset should be properly established but, in general, could take into consideration: (a) set-up costs, manual vs. automated processing blood cultures, manual for the Mini-Lab; (b) labour, the simpler nature of InTray™ cassettes allows for greater savings in labour costs; (c) rent of space/facilities, for the Mini-Lab not yet identified as so far deployed only in MSF operations. However, current estimations of specimen costs in the Mini-Lab by using the InTray cassettes fall not far off estimations considered still high for low resource settings [25].

## 5. Conclusions

The advantages of using InTray™ cassette devices are numerous: they show good performance, are easy and safe to use, easy to stock and have a long shelf-life. The combination of the MH chocolate and Colorex Screen InTray™ cassettes is a valid alternative to classic media for the detection of pathogens from bloodstream infections, in particular in low-resource settings where classic media preparation could be challenging.

However, other than developing tropicalised and customised solutions, in-vitro diagnostics manufacturers should also focus in parallel on making them more cost-effective for LMICs; otherwise, they will remain accessible only in a few privileged conditions.

## Figures and Tables

**Table 1:** Organism distribution retrieved from blood cultures during the clinical evaluation study.

Organism	N° of Strains
*Staphylococcus aureus*	21
*Staphylococcuss epidermidis*	10
*Klebsiella pneumoniae*	9
*Pseudomonas aeruginosa*	7
*Enterobacter cloacae*	5
*Escherichia coli*	3
*Staphylococcus hominis*	3
*Staphylococcus capitis*	2
*Staphylococcus haemolyticus*	2
*Klebsiella oxytoca*	1
*Micrococcus luteus*	1
*Morganella morganii*	1
*Staphylococcus pettenkoferi*	1
*Streptococcus agalactiae*	1
*Streptococcus constellatus*	1
*Streptococcus mitis*	1
*Streptococcus pneumoniae*	1
Total	70

**Table 2:** Microbial types of mixes used for the study.

Types of Mixes	Combination
Clinically relevant	*Acinetobacter baumannii—Klebsiella pneumoniae*
	*Escherichia coli—Streptococcus pneumoniae*
	*Enterococcus faecalis—Escherichia coli*
	*Enterococcus faecalis—Staphylococcus aureus*
	*Klebsiella pneumoniae—Streptococcus pneumoniae*
Colony colour challenge	*Pseudomonas aeruginosa—Salmonella* spp.
	*Staphylococcus aureus—Staphylococcus epidermidis*
	*Klebsiella pneumoniae—Enterococcus faecalis*
Contaminants	*Escherichia coli—Candida albicans*
	*Escherichia coli—Staphylococcus epidermidis*
	*Enterobacter cloacae—Corynebacterium striatum*

**Table 3:** Results of the reference validation study expressed in terms of the percentage of positivity at 24 h and 48 h of incubation.

	Total (n)	Growth on Both Agars	InTray Only	Reference Only	Percentage of Positive InTrays after 24 h Incubation	Percentage of Positive InTray after 48 h Incubation
All other bacteria/yeast *	433	433	0	0	100%	na
*Streptococcus* *pneumoniae*	49	47	0	2	98.0%	100%
*Streptococcus* *pyogenes*	8	6	0	2	75.0%	90%
Total	490	486	0	4	99.2%	

* *Burkholderia cepacia*, *Escherichia coli*, *Klebsiella pneumoniae*, S*almonella Typhimurium*, *Staphylococcus aureus*, *Haemophilus influenzae*, *Neisseria meningitidis*,* Candida tropicalis*, *K. oxytoca*, *E. cloacae*, *C. freundii*,* Salmonella Typhi*, *P. aeruginosa*, *A. baumannii*, *S. maltophilia*, *S. agalactiae*,* S. suis* and *Enterococcus faecalis*.

**Table 4:** Results of the clinical evaluation study expressed in terms of percentage of positivity.

	Total (n)	Growth on Both Agars	InTray Only	Reference Only	Percentage of Positive InTray
All other bacteria/yeast *	66	66	0	0	100%
*Streptococcus* *pneumoniae*	1	0	0	0	0%
Other *Streptococcus* spp **	3	2	0	0	66%
Total	70	68	0	0	97.1%

* *E.cloacae*,* E.coli*, *K. pneumoniae*, *K. oxytoca*, *M. luteus*, *M. morganii*,* P. aeruginosa*, *S. aureus*, *S. capitis*, *S. epidermidis*,* S. haemolyticus*, *S. hominis*, *S. pettenkoferi*. ** *S. constellatus*, *S. mitis*, *S. agalactiae*.

**Table 5:** Table of discrepancies of growth on InTray MH chocolate with reference media, including the number of isolated colonies. n/a = information not available.

	N of Strains	Time to Detection	N of Isolated Colonies	N of Colonies on Reference	Growth Characteristics	Discordance with Reference (Y, N)
*E. coli*	2	16 h	No isolated colonies	>10	n/a	N
*M. luteus*	1	20 h	>10	>10	Much smaller colonies on InTray	Y
*M. morganii*	1	16 h	No isolated colonies	>10	n/a	N
*P. aeruginosa*	3	16 h	No isolated colonies	>10	n/a	N
*S. aureus*	2	20 h; 16 h	>10	>10	Presence of white colonies also on reference	N
*S. capitis*	2	20 h	1–10; >10	>10	n/a	N
*S. epidermidis*	5	20 h	>10	>10	One strain with very faint growth	Y
*S. hominis*	1	16 h	>10	>10	two types of colonies also on reference	N

**Table 6:** Detection of mixed cultures and presence of single colonies on InTray MH chocolate and Colorex Screen media cassettes. * the total number of mixed cultures included only the number of replicates of mixes successfully recovered from the reference media (in bold in the table).

Types of Mixes		Detection of Mixed Culture on InTray (Comment, n of Mixes Replicates)		Presence of Single Colonies on InTray(Comment, n of Single Replicates)	
		Colorex Screen	MH Chocolate	Colorex Screen	MH Chocolate
Clinically relevant	***Acinetobacter baumannii*** ***Klebsiella pneumoniae***	3/3	Possible but difficult to detect based on morphology only, 3/3	2/9 Predominance of *K. pneumoniae*	Mixed layers, 0/9 Mixed layers, 0/9
	*Escherichia coli* *Streptococcus pneumoniae*	No growth of *S. pneumoniae* also on reference, 0/3	No growth of *S. pneumoniae* also on reference, 0/3	9/9 No growth (0/9)	9/9 No growth (0/9)
	***Enterococcus faecalis*** ***Escherichia coli***	3/3	Size of colonies was different on some InTrays, 1/3	9/9 9/9	3/9 Overgrowth, 9/9
	***Enterococcus faecalis*** ***Staphylococcus aureus***	3/3	Not possible to detect based on morphology only, 0/3	9/9 7/9	6/9 6/9
	*Klebsiella pneumoniae* *Streptococcus pneumoniae*	No growth of *S. pneumoniae* on reference, 0/3	No growth of *S. pneumoniae* on reference, 0/3	9/9 No growth, 0/9	9/9 No growth, 0/9
Colony colour challenge	***Pseudomonas aeruginosa*** ***Salmonella*** **spp.**	2/3	Possible but difficult to detect based on morphology only, 1/3	3/9 3/9	3/9 3/9
	***Staphylococcus aureus*** ***Staphylococcus epidermidis***	3/3	Not possible to detect based on morphology only, 0/3	9/9 9/9	9/9 9/9
	***Klebsiella pneumoniae*** ***Enterococcus faecalis***	3/3	3/3	9/9 9/9	8/9 9/9
Contaminants	*Escherichia coli* *Candida albicans*	No growth of *C. albicans* on reference, 0/3	No growth of *C. albicans* on reference, 0/3	9/9 No growth, 0/9	9/9 No growth, 0/9
	*Escherichia coli* *Staphylococcus epidermidis*	No growth of *S. epidermidis* on reference, 0/3	No growth of *S. epidermidis* on reference, 0/3	9/9 No growth, 0/9	9/9 No growth, 0/9
	*Enterobacter cloacae* *Corynebacterium striatum*	No growth of *C. striatum* on reference, 0/3	No growth of *C. striatum* on reference, 0/3	9/9 No growth, 0/9	9/9 No growth, 0/9
Tot mixed infections included * = 54 Tot grown strains = 162		51/54 (91.4%)	24/54 (44.4%)	132/162 (81.5%)	110/162 (67.9%)

**Table 7:** Table of discrepancies between InTray Colorex Screen and reference media for polymicrobial cultures. Expected colours are either from the manufacturer IFU (* Biomed Diagnostics), or as shown on reference media.

	Time to Detection (h)	Expected Colour	Colony Colour on Colorex	Colony Aspect on MH Chocolate	Discordance with Reference (Y, N, Description)
*Acinetobacter* *baumannii*	16 h	White beige	White	Difficult to distinguish, small and wrinkled colonies	Y mix clearly visible on reference compared to Colorex Screen
*Enterobacter cloacae*	16 h	Metallic blue *	Blue	Round with irregular margins	N
*Enterococcus faecalis*	16 h	Teal blue *	Blue	Round and small	N
*Escherichia coli*	16 h	Dark pink/reddish *	Pink	Round, smooth colonies	N
*Klebsiella pneumoniae*	16 h	Metallic blue *	Blue	Round, smooth colonies	N
*Pseudomonas aeruginosa*	16 h	Cream, translucid *	White/cream	Round with irregular margins	N
*Salmonella* spp.	16 h	White	White/light pink	Round small with regular margins	N
*Staphylococcus aureus*	16 h	Golden, small, matt *	White/gold	Round small with regular margins	N

**Figure A1:**
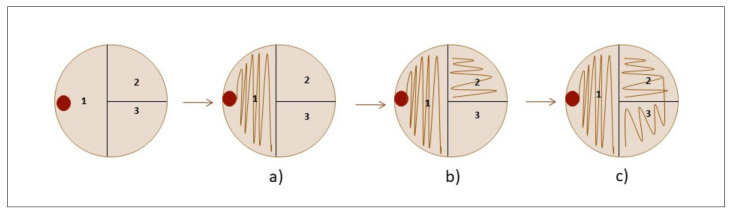
Inoculation technique for InTray cassettes: (**a**) expand the drop by streaking on half the surface (1, first quarter); (**b**) streak on a new quarter (2, second quarter) without touching the previous one; (**c**) repeat on the last quarter (3, third quarter) by touching the previous one.

**Figure A2:**
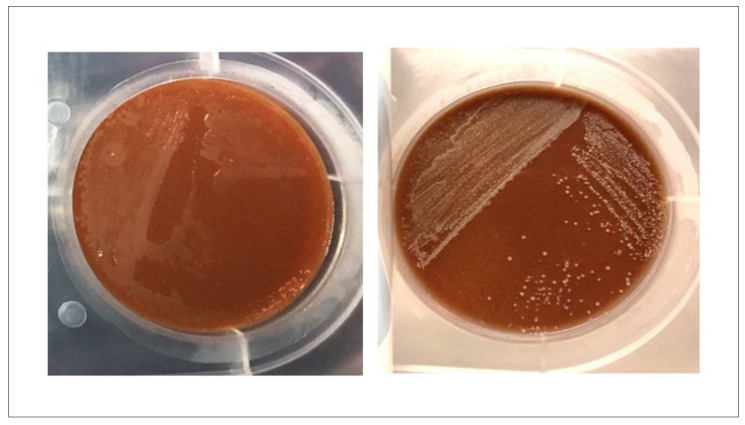
Comparison of subcultures of *S. aureus* on InTray MH Chocolate agar before (**left**) and after (**right**) optimisation of InTray subculturing protocol.

## Data Availability

Not applicable.

## Appendix A

During a proof-of-concept study, different InTray™ cassettes (Biomed Diagnostics Inc., OR, USA) agar compositions were tested against reference media. Isolation of single colonies was one of the indicators for ease of use, but we found that when the preparation of the InTray cassettes was performed as recommended by the manufacturer, an excess of condensation inside the cassettes could cause overgrowth on the agar surface with few single colonies. We, therefore, optimized, by making small adjustments, to both the preparation and inoculation steps.

Preparation of the InTray cassette was done as follows: (a) after the cassettes were removed from the fridge, they were tilted to concentrate the liquid on one side of the agar chamber, (b) the protective seal of the chamber was carefully removed while holding the cassette tilted, (c) the liquid on the side was carefully withdrawn with a sterile pipette; (d) the cassette was then resealed using the external protective label; (e) the cassette was incubated to dry out in upward position in an incubator at 35 ± 1 °C for at least 1 h. Drying time was defined based on the procedure routinely used in the research laboratory by technicians pouring standard plates.

Inoculation on the InTray cassettes was performed using a Safety Subculture Unit (SCU) (ITL BioMedical, Reston, VA, USA). The SCU offers many advantages; other than avoiding needlestick injuries, it also has a transparent plastic tip to visually control the blood flow during subculturing and a filter cap to safely exhaust the gas produced by growth inside positive blood cultures when pricking the bottle cap. After the drop was deposited on the InTray cassettes, a 1 µL sterile loop was used to streak the sample with a variation of the classic streaking method as follows; (a) streak the drop on half of the surface with a 1 µL loop; (b) repeat streaking on a new quarter of the InTray with the same loop, without touching the first quarter, to completely unload the loop; (c) repeat striking on the last quarter with the same loop, this time touching the last lines of the previous quarter. (Figure A1)

These small adjustments to the protocol improved the growth of single isolated colonies on InTray cassettes. The Figure below shows growth on InTray before and after protocol optimization. (Figure A2).

## Appendix B

For optimal growth, some fastidious organisms such as *Streptococcus* spp., *Neisseria spp* and *Haemophilus* spp. etc., require a 5% CO_2_ atmosphere. Based on the InTray™ cassette technology, Biomed Diagnostics has developed and validated a self-contained system for the production of carbon dioxide, the InTray GC. The InTray GC device consists of a rectangular tray with an inner well containing any agar formulation. Since in a previous proof of concept study, results showed that the growth of some *Streptococcus* species was improved by incubation in a normal air incubator, we designed a small test comparing the normal InTray cassettes incubated in a normal incubator or in a CO_2_ incubator, versus the InTray GC device. In this small study, 3 fastidious species (1 *Haemophilus influenzae*, 1 *Streptococcus pneumoniae*, and 1 *Neisseria subflava*), 2 non-fermenters (1 *Acinetobacter baumannii*, 1 *Pseudomonas aeruginosae*) and 1 *Klebsiella pneumoniae* were cultured from clinical samples; 1 ATCC *H. influenzae* was used as control. The clinical strains and the control strain were cultured overnight in blood culture bottles and then subcultured in triplicate under the 3 growth conditions: on InTray MH chocolate agar with normal incubator, on InTray GC MH chocolate with normal incubator and on InTray MH chocolate agar incubated in a CO_2_ incubator. The incubation temperature was 35 ± 1 °C, and growth was recorded after 16 h, 24 h and 48 h incubation. The results are shown in the table below (Table A1).

**Table A1:** Cumulative growth of all strains on InTray Chocolate Agar at different times of growth (N = 19).

	Cumulative Growth (N InTray, %)		
Time of Growth	CO_2_ Incubator		Air Incubator
	InTray GC MH Chocolate	InTray MH Chocolate	InTray MH Chocolate
16 h	32 (56%)	42 (74%)	45 (79%)
24 h	54 (95%)	57 (100%)	57 (100%)
48 h	57 (100%)	57 (100%)	57 (100%)

After 48 h of incubation, all strains grew on all InTray cassettes and incubating conditions. The integrated CO_2_ device doesn’t seem to confer any advantage on growth, instead causing a slower growth rate for certain organisms, in this case, the two non-fermenting species. In the case of incubation in a normal air incubator, in contrast, after 16 h, growth was visible in 45 compared to the 42 plates incubated in the CO_2_ incubator, suggesting a comparable or even faster growth under air conditions. Single colonies were visible in 100% of InTray MH chocolate, both incubated in CO_2_ and in an air incubator, compared to 93% of the InTray GC device. In conclusion, the InTray MH chocolate agar (without the CO_2_ device) incubated in a normal air incubator was the optimal combination for the growth of fastidious and non-fermenting organisms.

## Appendix C

**Table A2:** List of bacterial species used in the reference validation study and their origin.

Bacterial/Yeast Species Tested	Origin
*Acinetobacter baumannii*	DRC, Ecuador
*Burkholderia cepacia*	DRC, Ecuador, Benin
*Candida tropicalis*	DRC, Burkina Faso
*Citrobacter freundii*	DRC, Cambodia
*Enterobacter cloacae*	Ecuador, Benin, Cambodia
*Enterococcus faecalis*	DRC, Benin
*Escherichia coli*	DRC, Burkina Faso, Cambodia, Ecuador, Benin
*Haemophilus influenzae*	Burkina Faso, France, Belgium
*Klebsiella oxytoca*	Benin, Ecuador
*Klebsiella pneumoniae*	DRC, Benin, Burkina Faso, Cambodia
*Neisseria meningitidis*	Burkina Faso
*Pseudomonas aeruginosa*	DRC, Benin, Cambodia
*Salmonella Typhi*	Peru, DRC, Cambodia
*Salmonella Typhimurium*	DRC, Cambodia, Burkina Faso, Benin, Gambia
*Staphylococcus aureus*	DRC, Benin, Burkina Faso, Cambodia
*Stenotrophomonas maltophilia*	DRC, Cambodia
*Streptococcus agalactiae*	Cambodia
*Streptococcus pneumoniae*	DRC, Burkina Faso, Cambodia
*Streptococcus pyogenes*	DRC, Burkina Faso, Cambodia
*Streptococcus suis*	Cambodia
